# Pharmacogenetic Variation in Over 100 Genes in Patients Receiving Acenocumarol

**DOI:** 10.3389/fphar.2017.00863

**Published:** 2017-11-23

**Authors:** Vanessa Gonzalez-Covarrubias, Javier Urena-Carrion, Beatriz Villegas-Torres, J. Eduardo Cossío-Aranda, Sergio Trevethan-Cravioto, Raul Izaguirre-Avila, O. Javier Fiscal-López, Xavier Soberon

**Affiliations:** ^1^Instituto Nacional de Medicina Genomica, Mexico City, Mexico; ^2^Instituto Nacional de Cardiologia, Mexico City, Mexico

**Keywords:** coumarins, acenocumarol, pharmacogenomics, Mexican Mestizo, population differences, targeted sequencing

## Abstract

Coumarins are widely prescribed worldwide, and in Mexico acenocumarol is the preferred form. It is well known that despite its efficacy, coumarins show a high variability for dose requirements. We investigated the pharmacogenetic variation of 110 genes in patients receiving acenocumarol using a targeted NGS approach. We report relevant population differentiation for variants on *CYP2C8, CYP2C19, CYP4F11, CYP4F2, PROS*, and *GGCX, VKORC1, CYP2C18, NQO1*. A higher proportion of novel-to-known variants for 10 genes was identified on 41 core pharmacogenomics genes related to the PK (29), PD (3), of coumarins, and coagulation proteins (9) including, *CYP1A1, CYP3A4, CYP3A5*, and *F8*, and a low proportion of novel-to-known variants on *CYP2E1, VKORC1*, and *SULT1A1/2*. Using a Bayesian approach, we identified variants influencing acenocumarol dosing on, *VKORC1 (2), SULT1A1 (1)*, and *CYP2D8P (1)* explaining 40–55% of dose variability. A collection of pharmacogenetic variation on 110 genes related to the PK/PD of coumarins is also presented. Our results offer an initial insight into the use of a targeted NGS approach in the pharmacogenomics of coumarins in Mexican Mestizos.

## Introduction

Anticoagulants such as warfarin, acenocumarol, and phenprocoumon act as inhibitors of vitamin K reducing enzymes, which regenerate vitamin K, a cofactor for several clotting proteins. Acenocumarol is the coumarin of choice in Europe and Latin America (Ufer, 2005). The heterogeneity in coumarins efficacy, safety, and dosing has been partly explained by clinical, demographic, and genetic parameters. Several polymorphisms on *CYP2C9, VKORC1*, and *CYP4F2* can account for about 40–50% on coumarin dose differences (Scott et al., 2014). However, most studies have been performed for warfarin and in populations other than Mexican. Acenocumarol (4-nitrowarfarin) is the most commonly prescribed oral coumarin in the public health care system in Mexico. In contrast to warfarin, the more potent isomer, S-acenocumarol, is rapidly eliminated and the drug's therapeutic effect is most likely due to R-acenocumarol. The R isomer is metabolized by several members of the cytochrome P-450 family including, CYP2C9, CYP2C19, CYP2C8, CYP2C18, CYP3A4, CYP1A1, and CYP1A2 (Tassies et al., 2002; Ufer, 2005). Hence, genetic variation on genes coding for these proteins should putatively influence acenocumarol dosing.

The collection of pharmacogenetic variation in Mexican populations is still scarce (Fricke-Galindo et al., 2016). Reports indicate that some of the actionable markers on *VKORC1* and *UGT1A1* present significant population differences (Bonifaz-Peña et al., 2014), suggesting the existence of variants with distinctive allele frequency in these populations potentially influencing drug response.

Endeavors are currently ongoing to amass a more comprehensive picture of the pharmacogenetic variation in Mexican Mestizos. Here, we investigated genetic variation in over 100 genes by targeted NGS in patients receiving acenocumarol, including genes involved in general pharmacokinetics and pharmacodynamics, vitamin K recycling, and coagulation proteins, these latter also potentially affecting acenocumarol response (Allan et al., 2005; Harrington et al., 2008; Carcao et al., 2015; Tong et al., 2016). Clinical and genetic data were used to develop an algorithm to explain dose variability in this group of patients.

Genomic data analyses provided with a collection of pharmacogenetic variation for this population. This approach hints to toward the consideration of multiple variants to assess acenocumarol dosing for an individualized dose assessment.

## Materials and methods

Participants and DNA extraction. The National Institute of Cardiology in Mexico City prescribes acenocumarol on a regular basis mostly after stroke, stent implants, or for thrombosis. A hundred and fifty patients treated with acenocumarol between 2006 and 2010 were surveyed and monitored for acenocumarol efficacy through at least three consecutive INR measurements. Of these, 103 blood samples were available for DNA extraction using the DNeasy Blood & Tissue kit (Qiagen, Valencia CA, USA) from a routine blood sample in EDTA-Vacutainer collection tubes, sample characteristics are depicted in Table 1. All participants gave written informed consent according with the Declaration of Helsinki. The project was reviewed and approved by The Research and Ethics Committees at The National Institute of Cardiology and The National Institute of Genomic Medicine (INMEGEN) Mexico City, project approval 25/2016/I.

**Table 1:** Patient demographics.

	**Males**	**Females**	***P*****-value**
***N***	**46**	**54**	
Age (y)	54 (17-84)	56 (26-85)	>0.05
Weight (kg)	76 (54-121)	64 (37-90)	<0.05
Height (m)	1.7 (1.5-1.8)	1.5 (1.4-1.7)	<0.05
INR	2.8 (2.1-3.4)	2.8 (2.0-3.9)	>0.05
Dose (mg/wk)	15.4 (3.2-56)	15.0 (4.0-52)	>0.05
Mo under treatment	12 (4-60)	13 (6-56)	>0.05

### Next generation sequencing

We investigated coding, 25 bp of adjacent introns, and 5′ and 3′ UTR regions of 110 genes related to general pharmacogenomics including core pharmacokinetics and pharmacodynamics targets in 100 DNA samples. We selected these genes according to the general PGx and the PK/PD of acenocumarol by searching the available literature using the keywords, pharmacogenetics, pharmacogenomics, acenocumarol, coumarin pharmacokinetics, and pharmacodynamics (van Leeuwen et al., 2008; Soria et al., 2009; Whirl-Carrillo et al., 2012; Tong et al., 2016). Regions of interest were captured using a Haloplex custom Target Enrichment System (Agilent Technologies, Santa Clara, CA, USA) defined for 150 × 2 paired-end reads, in a panel size of 1.1Mbp. In addition, we included a set of 360 ancestry informative markers (AIMS) to assess genetic admixture using SNPs from the HapMap database for CEU and YRI populations, and Natives from Mexico (Galanter et al., 2012). Sequencing libraries were generated according to the manufacturer's protocol (version D.5, May 2013). Briefly, all 100 DNA samples (225 ng) were digested with 8-paired restriction enzymes, fragmentation pattern was analyzed in a 2100 Biolanalyzer (Agilent Technologies). DNA fragments were hybridized with the Haloplex synthetic probes, adapters were ligated followed by PCR amplification for library enrichment. Library quality for fragment size and molarity was also performed using 2100 Biolanalyzer information. Samples were pooled and sequenced in a Genome Analyzer II (Illumina, San Diego, CA, USA) according to the manufacturer's instructions. Targeted genes are listed in Supplemental Table ST1.

### Bioinformatic and statistical analyses

Sequence reads were processed according to the Broad Institute recommended best practices workflow and the Genome Analysis ToolKit (GATK) (Acland et al., 2013; Van der Auwera et al., 2013). Briefly, paired-end reads were trimmed to remove adapters and low quality regions using Trimmomatic (Bolger et al., 2014), reads with an average *Q* ≤ 30 were discarded, followed by elimination of reads shorter than 36 nucleotides. Mapping and alignment of sequencing reads were performed with BWA, Samtools, and Picard using the hg19 human genome reference (dbSNP build 137) (McKenna et al., 2010). Base quality score calibration and single nucleotide variant (SNV) calling were assessed using GATK v3.3. Variants were confirmed visually in the integrative genomic viewer, IGV (Robinson et al., 2011), and their functional impact was annotated using SnpEff, and ranked as low, moderate, modifier, or high (Sherry et al., 2001; Cingolani et al., 2012; Exome Variant Server^1^).

The data analysis toolset, PLINK was used to determine descriptive statistics, allele frequencies, Hardy-Weinberg, and population differentiation, this latter was assessed by determining the measure of genetic variance in this subpopulation relative to population variance in other continental groups using the *F*_*ST*_ statistic. A threshold value of *P* < 0.05 after FDR was considered as statistically significant (Purcell et al., 2007).

### Pharmacogenetic model

We utilized a Bayesian statistical approach to incorporate genetic variants to an algorithm for acenocumarol dose estimation (Sebastiani et al., 2009; Chen et al., 2012). The rejection of the null hypothesis (lack of association between acenocumarol dose and genetic variants) was based on probabilities of stochastic computations of Markov Chain Monte Carlo methods (MCMC). Also, we tested the association between dose and all SNV alone or in combination including those previously identified via single-SNP analysis (*P* > 0.05, FDR). We considered 4614 SNPs and 815 variant interactions for model development. This strategy allows for the identification of independent genetic variants or those that depend on each to influence dose variation. Variants were considered with a MAF <0.95 and >0.05, and interactions between variants within the same gene with frequencies <0.9 and >0.10, i.e., that two or more variants in a gene may have adding or balancing effects on the dose. First, we used a Bayesian Generalized Linear model for variable selection to obtain the posterior probability of a gene variant affecting acenocumarol dose, then we used Bayes Factors, a form of Bayesian hypothesis tests, to prioritize a set of models, and then we evaluated the selected models through Deviance Information Criterion (DIC), a measure of model selection related to AIC and BIC criteria, commonly used in Bayesian hierarchical models. Briefly, for the former, we used a gamma likelihood function with logarithmic link function, variance τ and mean conforming to Equation (1), where *v*_1, *j*_ and *v*_2, *j*_ represent binary variables for each genotype of a SNP*j*, refers to interactions between variants, G is a set of genes g, and ng the number of SNPs in g; *v*_*r*,*j*_*g*__ represents genotype r of SNP j in gene g, and $\beta_{j_{g},k_{g}}^{r_{1},r_{2}}$ represents the effect size of genotypes *v*_*r*_1_, *j*_*g*__ and *v*_*r*_2_, *k*_*g*__; Ij and Ij,k are binary variables for the inclusion or exclusion of SNPs and SNP interactions, and *x*_*i*_ and α_*i*_ represent m non-genetic covariates including age, sex, BMI, and height.

(1) $$\begin{array}{l} \log\left(\text{E}\left[\text{y|}\theta \right]\right)=c+\sum_{i}^{m}\alpha_{i}x_{i}+\sum_{j}^{n}I_{j\left(\beta_{1,j}v_{1,j}+\beta_{2,j}v_{2,j}\right)}\\ \;+\sum_{g\in G}\sum_{j_{g}<k_{g}}^{n_{g}}I_{j_{g},k_{g}}(\beta_{j_{g},k_{g}}^{1,1}v_{1,j_{g}}v_{1,k_{g}}+\beta_{j_{g},k_{g}}^{1,2}v_{1,j_{g}}v_{2,k_{g}}\\ \;+\beta_{j_{g},k_{g}}^{2,1}v_{2,j_{g}}v_{1,k_{g}}+\beta_{j_{g},k_{g}}^{2,2}v_{2,j_{g}}v_{2,k_{g}}) \end{array}$$

Where, τ ~ *Gamma* (λ, κ), $c\sim Normal\;\left(0,\;\tau_{\mu }^{2}\right)$, $c\sim Normal\;\left(0,\;\tau_{\mu }^{2}\right)$, $\beta_{j.k}\sim Laplace\left(0,\;\tau_{\beta }^{2}\right)$, $\beta_{j_{g},k_{g}\;}^{r_{1},\;r_{2}}\sim Laplace\;\left(0,\;\tau_{\beta }^{2}\right)$, *I*_*j*_ ~ *Bernoulli*(π), *I*_*j*_*g*_, *k*_*g*__ ~ *Bernoulli*(π), π ~ *U*(*a, b*), and $\alpha_{i}\sim Normal\left(0,\;\tau_{\alpha }^{2}\right)$

Next, we standardized clinical variables for mean zero and unitary variance, and using JAGS 4.1.0 and R 3.2,0 we obtained MCMC from the posterior distribution. We ran five chains of 110,000 iterations each, including a burn-in period of 10,000 iterations and random initial values, convergence was verified via the Gelman-Rubin statistic $\hat{R}<1.2$, followed by a series of Bayes Factors to condition on the presence or absence of variants, branching them into a decision tree, as part of the pharmacogenetic model development. Further details on the model development were included in Supplemental Table ST2.

## Results

Demographic characteristics stratified by sex are presented in Table 1. Bioinformatic analyses revealed 5108 variants in 110 genes in 100 DNA samples with an average depth of 250x and >98% coverage, but a wide range was registered depending on the gene (30x−600x, 80–100%). These 110 genes represent less than 1% of the coding genome, approximately 25% of a pharmacogenome, more than half of the Coriell reference list for pharmacogenomics, and include >20% of actionable pharmacogenetic markers listed by CPIC (Pratt et al., 2010; Relling and Klein, 2011). After quality control, variant calling, and annotation, 4290 SNVs were utilized for statistical analyses (ST3). There was a complete agreement between genotypes of variants assessed by NGS and allele discrimination performed for CYP2C9^*^2,^*^3, and ^*^5, CYP4F2 rs2108622, and *VKORC1* rs9934438. Admixture analysis with 314 ancestry informative markers showed an average population structure of 50–92% Mexican Native and 6–54% Caucasian (CEU), all individuals showed less than 5% of Sub-Saharan African (YRI) admixture.

Of these 4290 SNVs, 28% have not been reported before (1237 without an rs identifier) and 274 of these novel variants had a minor allele frequency (MAF>1%). On average, each individual showed 908 SNVs, 534 heterozygous and 374 homozygous, of which 258 were present per individual (Table 2). Four-hundred and seven variants in 65 genes did not suffice the equilibrium of Hardy-Weinberg (9.8%, Supplemental Table ST4).

**Table 2:** Summary of NGS genetic variation.

	***N***	**Observations**
Total SNVs	4,290	in 100 individuals
SNVs per individual	908	534 heterozygous, 374 homozygous
Coding	3,335	
UTRs	471	
Non coding	484	
Known SNVs	3,053	650 per individual (384 heterozygous and 266 homozygous)
Novel SNVs	1,237	258 per individual (150 heterozygous and 108 homozygous)
Novel SNVs MAF>1%	274	Present in 56 genes including 19CYPs, 3 UGTs, *SULT1A2*
Heterozygous SNVs	2,463	1660 known and 803 novel
Heterozygous, high impact	27	8 novel variants on 8 genes including *CYP3A4* and *CYP2B6*, and 19 known variants in 9 CYPs, 2 UGTs, *SULT1A1*, and 5 coagulation proteins
Heterozygous, moderate impact	325	106 novel in 56 genes including 12 CYPs, 5 UGTs, and *SULT1A2*, 219 known variants in 69 genes, including, 14 CYPs, 6 UGTs, and *SULT1A2*
Total homozygous	1,110	Known and novel variants
Homozygous, high impact	9	Present on 4 CYPs, including, *CYP2B6* POS.4151213, *CYP2C9* rs114071557, and on *UGT2B7*
Homozygous, moderate impact	129	36 novel variants in 25 genes (7 CYPs, 3 UGTs) including *CYP4F2* POS15996832, *CYP2D6* POS42522724, and 93 known variants in 33 genes (12 CYPs, 5 UGTs, and 5 drug targets)

*HGVS, Human Genome variation society nomenclature; C, coding region; P, protein. The 1000 Genomes Project Consortium et al. (2015). POS indicates the position on the chromosome for novel variants*.

Variants were classified by SNPEff according to their to their *in-silico* functional impact as high, moderate, modifier, or low (Cingolani et al., 2012). We listed a total of 36 known SNVs (27 heterozygous and 9 homozygous) with a high functional impact (Table 2 and ST3). These resequencing descriptive statistics seem to compare to other reports (Waldron, 2016).

### Pharmacogenetic variation

The *F*_*ST*_ statistic was assessed to evaluate genetic differentiation between Mexican Mestizos and three major continental populations, Chinese Han from Beijing (CHB), Yoruba from Ibadan, Nigeria (YRI), and Europeans from Utah, USA (CEU) utilizing the 1000 genomes database. YRI showed the largest differentiation with 377 variants with a *F*_*ST*_ value above 0.25, followed by CEU (51 variants with *F*_*ST*_ > 0.25), and CHB (32 variants with *F*_*ST*_ > 0.25, ST5). *F*_*ST*_ >0.25 values were identified for variants on several genes related to the coagulation cascade or coumarin metabolism. For example, when comparing to Caucasians we found high population differentiation for variants on *CYP2C8, CYP2C19, CYP4F11, CYP4F2, PROS*, and *GGCX*. Comparing to CHB, differences arose on *CYP2C8, VKORC1, 2C18, NQO1*, and for YRI differences were observed on major CYPs, FMOs, *F13B, F8, PROS*, and *SERPINA10* among others (ST5). Allele frequency comparisons between Mexicans from the 1000 genome project (MXL) and those in this study showed similar *F*_*ST*_ values for most variants, except for *CYP2C18* rs2281889, *CYP2C8* rs1891071, *CYP4F2* rs309319, and *CYP4F11* rs11086013, for which we observed *F*_*ST*_ values between 0.15 and 0.33.

We analyzed allele frequency variation for 30 major pharmacogenes and 10 genes related to the coagulation cascade. The largest number of variants per gene was observed on *SULT1A1, CYP2E1, CYP1B1, CYP3A4, CYP3A5, F5*, and *F11*. Interestingly, *CYP1A2, CYP3A4, CYP3A5, CYP1A6, F11, F13B*, and *F8 s*howed a large proportion of novel variants compared to known variants. Genes with significantly fewer variants were, *CYP1A3, CYP1A5, CYP1A9*, and *F9*, the three former did not show any novel variants (ST3). Next, we assessed the presence of known and novel variant considering a MAF >5% and a predicted functional impact as high or moderate in these genes. For known variants, we list 7 with a high predicted functional impact, 2 on UGTs and one on *SULT1A1, CYP2C9, CYP2C19*, and *CYP2C8* (Table 3).

**Table 3:** Novel and known variants on relevant pharmacogenes at MAF > 5%.

**Gene**	**Chr**	**ID**	**Predicted functional impact**	**HGVS_C**	**HGVS_P**
*UGT1A10*	2	rs200144439	High	c.1533C>A	p.Tyr511^*^
*UGT2B7*	4	rs11935951	High	c.−159+1G>T	Intron/splicing
*CYP2C19*	10	rs28399504	High	c.1A>G	p.Met1?
*CYP2C9*	10	rs114071557	High	c.1A>G	p.Met1?
*CYP2C8*	10	rs181982392	High	c.1198G>T	p.Glu400^*^
*SULT1A1*	16	rs144422872	High	c.307C>T	p.Arg103^*^
**NOVEL VARIANTS**					
**Gene**	**Chr**	**Position**	**Predicted functional impact**	**HGVS_C**	**HGVS_P**
*F5*	1	169530002	Moderate	c.376G>T	p.Ala126Ser
*F10*	13	113803697	Moderate	c.1333C>T	p.Arg445Cys
*UGT1A10*	2	234676866	Moderate	c.1076G>T	p.Gly359Val
*UGT1A10*	2	234676884	Moderate	c.1094C>A	p.Ala365Asp
*SULT1A2*	16	28603787	Moderate	c.473C>A	p.Thr158Asn
*CYP3A5*	7	99250198	Moderate	c.1231C>T	p.Pro411Ser
*CYP3A4*	7	99358497	Moderate	c.1361T>C	p.Leu454Pro
*CYP3A4*	7	99365982	High	c.665C>A	p.Ser222^*^
*CYP3A4*	7	99377662	Moderate	c.118G>T	p.Gly40Trp
*CYP2C19*	10	96522531	Moderate	c.69C>A	p.Ser23Arg
*CYP2C8*	10	96800713	Moderate	c.896T>A	p.Phe299Tyr
*CYP2C8*	10	96802829	Moderate	c.967G>T	p.Val323Phe
*CYP2E1*	10	135352449	Moderate	c.1463G>T	p.Cys488Phe
*CYP1A2*	15	75042614	Moderate	c.535C>A	p.Leu179Met
*CYP4F2*	19	15996832	Moderate	c.1017G>T	p.Trp339Cys
*CYP2B6*	19	41518213	High	c.975C>A	p.Tyr325^*^
*CYP2D6*	22	42522724	Moderate	c.1346C>A	p.Ala449Asp
*NQO1*	16	69745184	Moderate	c.520A>C	p.Ser174Arg

*POS, position on the chromosome for novel variants. p.Met1?, change in the translation of the initiation codon with unknown effect*.

* *Insertion of a termination codon*.

**Figure 1:**
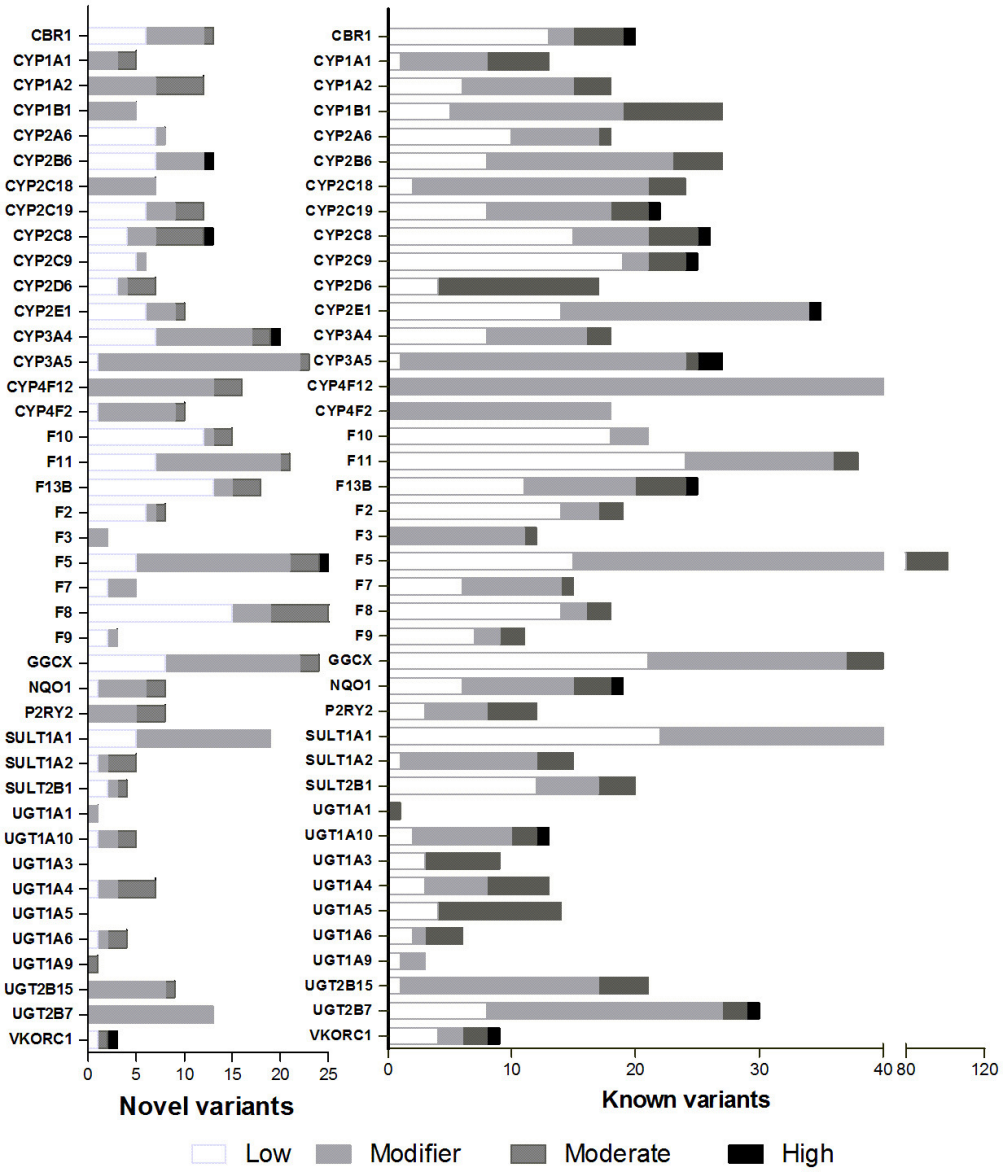
Proportion of novel and known variants. Functional impact determined *in-silico* is depicted in white for low impact, light gray for modifier, dark gray for moderate impact, and black for high impact.

Novel variants were observed on 37 of these 40 genes in counts from 1 on *UGT1A1*, to 23 on *CYP3A5*, 25 in *F*5, and 26 in *F8*. Of these, 2 showed a high functional impact predicting a stop codon on *CYP3A4* and *CYP2B6*, a moderate impact was reported for 16 variants on 14 genes (Table 3). The proportion of novel-to-known variants and its functional impact for these pharmacogenes is represented in Figure 1.

### Acenocumarol pharmacogenetic model

**Figure 2:**
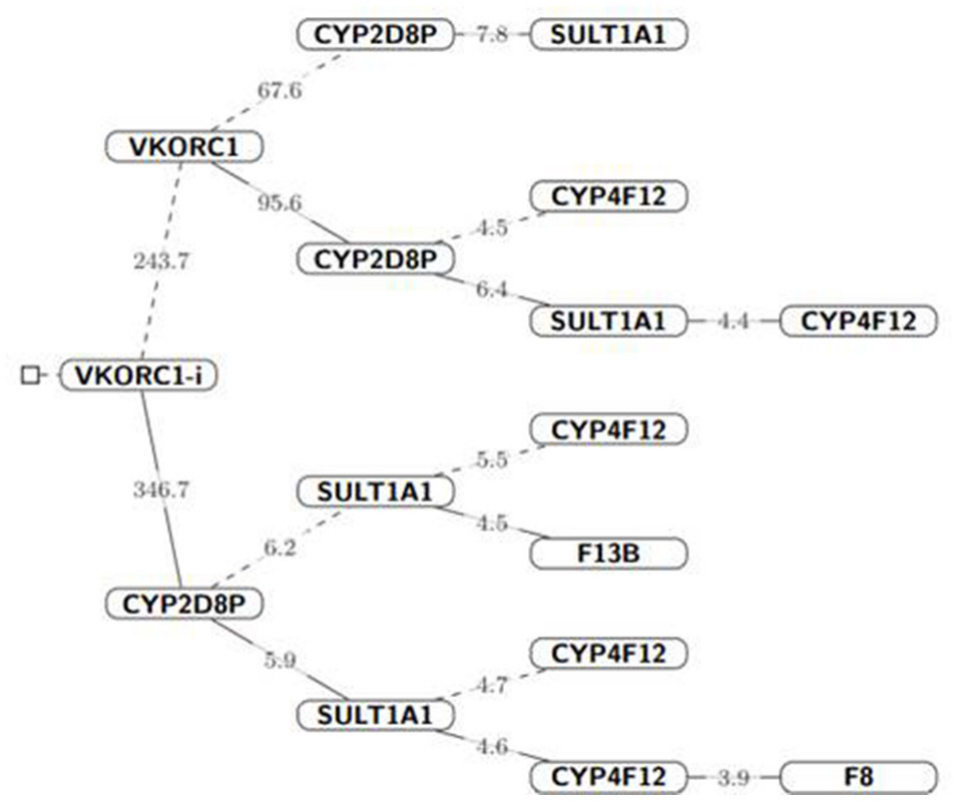
Hierarchical tree of variants influencing acenocoumarol dose. The parent node (solid lines) represent gene variants from which other variants depend for acenocouumaorl dose estimation, i.e., all probability statement in that branch are conditioned to the probabilistic event of the parent node (gene) *Iparent* = *1*, dashed lines indicate that the parent node was conditioned to zero, *Iparent* = *0*. The number on each line represents the Bayes Factor for the following branched off node; given the cut-off values. For visualization purposes, we did not condition on CYP2D8P, which was strongly associated with high-dose values (≥40 mg/wk) in 3 of the 6 patients receiving high doses. Nevertheless, both frequentist and Bayesian hypothesis testing suggested a strong association to coumarin dose for this variant (*P*-value < 0.05 and Bayes Factor > 100, respectively).

We developed a pharmacogenomic model to predict acenocumarol dose, using a Bayesian approach that included all SNP variants and the interaction among those on the same gene. We fitted the Bayesian GLM through five MCMC chains where genetic variants were prioritized by their posterior inclusion probability. The higher the posterior probability of a variant, the larger its influence on dose. Figure 2 shows a hierarchical tree indicating an ordered relevance of variants from *VKORC1, CYP2D8P*, and *SULT1A1*, followed by those on *CYP4F12, F13B*, and *F8*, Values of posterior probability for all variants are listed in ST7 and ST8.

The dosing algorithm accounted for age, sex, weight, and height. The interaction between *VKORC1* variants rs8050894 and rs9934438 which are in LD (*R* = 0.492), showed the highest posterior inclusion probability (mean, 0.96), followed by a novel variant, on *CYP2D8P* (POS42547668), and variants on *SULT1A1* rs11648192, and *CYP2C8* rs1058932, and rs2275620. Unfortunately, pharmacogenetic variant *VKORC1* rs9923231 did not pass NGS quality controls thus, it was not modeled, but it is in complete LD with rs9934438, which was included in these analyses (Rieder et al., 2005).

Final model evaluation, we used R to implement the series of Bayes Factors as described in Supplemental Table ST2 with a cut-off value, *c*_*j*_ = 3+*m*_*j*_, where *m*_*j*_ is the number of variants conditioned to be absent from the model, and 3 as a minimum cut-off value based on Harold Jeffrey scale of interpretation for Bayes Factors (Baldi and Long, 2001). We selected two models according to the lowest DIC values, the first one included SNVs interactions, *VKORC1* rs8050894 and rs9934438 and variants on *SULT1A1* and *CYP2D8P (ST7)*. The second model excluded variant interactions. Modeled variants and clinical parameters (age, sex, weight, and height) explain up-to 55.9% of dose variation for this study group. Values of high density intervals (HDI) are presented considering for 95% posterior probability (ST7 and ST8). The addition of additional variants to the model increased DIC values significantly which translates into a decreasing impact on acenocumarol dose (Table 4).

**Table 4:** Pharmacogenomic model parameters.

	**Genotype**	**μ**	**HDI**	**Bayes Factor^a^**
**MODEL 1**				
Intercept	–	0.884	0.72 *to* 1.03	>900
*SULT1A1*, rs11648192	C/T	0.203	0.06 *to* 0.33	91.3
*CYP2D8P*, POS42547668	T/C	0.899	0.54 *to* 1.27	>900
*VKORC1*, rs8050894, rs9934438	C/G, G/A	−0.213	−0.37 *to* −0.06	>900
*VKORC1*, rs8050894, rs9934438	G/G, A/A	−0.719	−0.97 *to* −0.48	>900
Age	–	−0.102	−0.17 *to* −0.04	23.48
Weight	–	0.047	−0.05 *to* 0.15	2.61
Height	–	0.110	0.01 *to* 0.22	22.08
Sex	–	−0.124	−0.33 *to* 0.05	6.41
**MODEL 2**				
Intercept	–	0.929	0.752 *to* 1.107	>900
*SULT1A1*, rs11648192	C/T	0.188	0.042 *to* 0.335	46.5
*CYP2D8P*, POS42547668	T/C	1.092	0.709 *to* 1.481	>900
*VKORC1*, rs9934438	G/A	−0.235	−0.399 *to* −0.061	>900
*VKORC1*, rs9934438	A/A	−0.529	−0.784 *to* −0.246	>900
Age	–	−0.099	−0.171 *to* −0.018	13.63
Weight	–	0.061	−0.037 *to* 0.17	2.37
Height	–	0.097	−0.021 *to* 0.211	10.54
Sex	–	−0.098	−0.313 *to* 0.092	3.43

a *The Bayes Factor corresponds to a hypothesis test with H_0_:β = 0 and H_1_:β = μ, where β is the coefficient and μ is the posterior mean. HDI is the high-density interval for μ*.

### Pharmacogenetic model considering variant interactions

Ln Dose = −0.6935–0.0071^*^age (y) + 0.0035^*^weight (kg) – 0.1136 (if male) + 1.0709^*^height (m) – 0.213 (if VKORC1 rs8050894 is C/G and rs9934438 is G/A) – 0.719 (if VKORC1 rs8050894 is G/G and rs9934438 is A/A) + 0.899 (if CYP2D8P POS.42547668 is T/C) + 0.203 (if SULT1A1 rs11648192 is C/T). Variance explained 55.9%.

### Pharmacogenetic model without variant interactions

Ln Dose = −0.5846 – 0.0069^*^age (y) + 0.0045^*^weight (kg) – 0.0945 (if male) + 0.9795^*^height (m) – 0.239 (if VKORC1 rs9934438 is G/A) – 0.529 (if VKORC1 rs9934438 is A/A) + 1.092 (if CYP2D8P POS. 42547668 is T/C) + 0.188 (if SULT1A1 rs11648192 is C/T). Variance explained 40.0%.

Finally, we used these models to recalculate acenocumarol dose in patients with an INR 2–3 receiving a stable dose. Pharmacogenetic dose calculations approached given acenocumarol doses for all INR-sable patients (*P* > 0.05) except for one patient, R4 who needed 5.4 mg/day, and models estimated 2.47 mg/day. All individual dose estimations were listed in ST9.

## Discussion

Here, we investigated pharmacogenetic variation in 110 genes by targeted NGS in patients treated with acenocumarol.

### Novel variants

Genetic variation analyses showed that the presence of novel variants varied widely among genes. For example, the largest number of novel variants (≥20) was observed on *CYP3A4, CYP3A5, SULT1A1, GGCX, F11, F5, F8*, and *F9*. High functional impact variants were present on *CYP2B6* (MAF, 50%), *CYP2C8* (MAF 1%), *CYP3A4* (MAF 8%), F5 (MAF 2%), and *VKORC1* (MAF 1%). These are relevant for its allele frequency, the dozens of drugs they metabolize, and because their impact predicts a stop codon. *CYP2B6* and *CYP3A4* are among the most polymorphic genes thus, it is not surprising the presence of relevant novel variants. Similarly, for *VKORC1* population differentiation has been previously reported and the presence of a novel variant with a high functional impact may be in part, a consequence of this stratification (Bonifaz-Peña et al., 2014). Novel variants on major metabolizing genes, *CBR1, CYP1A2, CYP3A4, CYP3A5, P2RY2*, and *UGT1A6* represented 40–50% of novel and known variants, suggesting that the collection of variation on these genes is probably not yet complete in Mestizos. Other metabolizing genes, *CYP1B1, CYP2C18, CYP2C19, UGT1A* members*, CYP2E1, SULT2B1*, and *SULT1A1* showed a low proportion of novel-to-know variants (Figure 1). This may not necessarily mean that genetic variation is complete for these genes. For example, *SULT1A1* presented 63 known and 19 novel variants ranking this gene as second with the largest number of variants.

Also, we confirmed population differences previously reported with an Fst > 0.19, on *VKORC1* rs9934438 and four variants on *UGT2B15* when comparing to YRI and CHB (Bonifaz-Peña et al., 2014). Differences between Mestizos and CEU were observed for *UGT2B15, CYP2E1, CYP1A2, CYP4F2, UGT2B7, F12*, and *F12* (ST5). Interestingly, a few variants showed an Fst > 0.20 between Mestizos from this study and Mexicans from Los Angeles (MXL) from the 1000 genome project, on *CYP2C8, CYP2C18*, and *CYP4F2* relevant for the pharmacokinetics of coumarins, phenytoin, vitamin K, and lipids. Allele frequencies of all variants are listed on ST6. Observations on these relevant pharmacogenes highlight the need to for a cautious implementation of pharmacogenomics in Mexican Mestizos.

We developed a pharmacogenetic model to estimate acenocumarol dose testing over four thousand variants. The model considered relevant variants on, *SULT1A1, CYP2D8P*, and *VKORC1*. For the latter gene, variants, rs8050894 and rs9934438, are well-known pharmacogenetic markers of coumarin dosing with the highest PharmGKB level of evidence. The interaction of these SNPs has already been reported as part of a haplotype (CG vs. TA) that aids to classify patients into high and low dose requirements (Rieder et al., 2005).

Interestingly, the model did not associate variants on *CYP4F2* or *CYP2C9* with acenocumarol dose (Table 4). Maybe because *CYP4F2* (rs2108622) has a lower impact (Danese et al., 2012), and R-acenocumarol is metabolized by several CYPs other than *CYP2C9*. Moreover, reported variants that impair CYP2C9 activity are present in low frequency in Mexican Mestizos (Villegas-Torres et al., 2015). Instead, we observed dose association with variants on *SULT1A1* and *CYP2DP8*. *CYP2D8P* is a pseudogene in the *CYP2D* cluster comprising *CYP2D6, CYP2D7P*, and *CYP2D8P* the former known to metabolize around 25% of all prescribed drugs. This cluster seems to have rapidly evolved due to environmental adversity with ethnic differences (Heim and Meyer, 1992). Wang et al identified a *CYP2D6* transcription enhancer in the *CYP2D* cluster supporting the consideration of variants outside the *CYP2D6* loci for functional genotyping (Wang et al., 2015). We identified a new variant on *CYP2D8P* POS.42547668, 26 Kbp upstream *CYP2D6*, and although there is no xenobiotic metabolism reported for this pseudogene, we can speculate that this variant is in LD with another one affecting gene expression or drug metabolism. The inclusion of many variants to dissect a pharmacogenetic phenotype is becoming more common as it increases our knowledge in paths and network interactions not previously considered (Cruz-Correa et al., 2017; Oliveira-Paula et al., 2017).

Our model is similar to others in that it includes typical clinical variants (age, sex, weight, and height), a dose prediction around 50% confidence, and the inclusion of *VKORC1* as the primary determinant of acenocumarol dose. And even though we report *VKORC1* rs9934438 vs. *VKORC1* 9923231 this latter, most commonly studied (Zhang et al., 2015; Tong et al., 2016) these are in complete LD. Finally, dose assessment using this model closely approached the dose received to achieve an INR 2–3, except for patient R4. Therefore, we delved into the genetic variability of this sample observing 20 heterozygous and 9 homozygous variants, these latter on *SULT1A1, FGB, CDH12, KCNJ6, CBR3*, and *CYP2E1*. However, this variation does not necessarily explain a lack of dose prediction. We can speculate that it is the presence of multiple variants on certain genes that affects several steps of the pharmacodynamics or pharmacokinetics and thus, drug efficacy.

We acknowledge the size and closed patient group studied retrospectively in individuals that were already assigned a dose by trial and error, not allowing for a prospective use of the genetic information. These observations will require confirmation and replication. We provide a list of 20 variants in 18 genes ordered by its impact on acenocumarol dose around the PK/PD of coumarins and the biochemistry of the coagulation cascade.

Our results offer an initial insight to the use of a genomic approach in pharmacogenetics showing that the advent of next generation sequencing may offer an alternative to identify and utilize individual variation to potentially explain a pharmacological relevant phenotype (Cheng et al., 2015). Future endeavors should focus on confirming these observations in a larger population.

As of June 2017, there are under a dozen reported studies of NGS in Mexican populations (NBCI), here, we present a list of variants in 110 pharmacogenes in Mexican Mestizos providing population information for allele frequency, differentiation from other continental groups and phenotype associations, which may complement the current catalog of pharmacogenomic variation in different populations.

## Author contributions

VG-C: Performed genomic experiments, interpreted results, and wrote the manuscript, JU-C: Analyzed the data and developed the Bayesian model, BV-T: Coordinated demographics and clinica data, performed genotyping experiments. JC-A: Conceived, planned, and performed the clinical study, ST-C: Conceived, planned, and performed the clinical study, RI-A: Conceived, planned, and performed the clinical study, OF-L: Conceived, planned, and performed the clinical study, XS: Conceived and coordinated genomic analyses.

### Conflict of interest statement

The authors declare that the research was conducted in the absence of any commercial or financial relationships that could be construed as a potential conflict of interest.

## Footnotes

^1^*Exome Variant Server. [updated 2014/01/02/10:04:37]*. Available online at: http://evs.gs.washington.edu/EVS

## Abbreviations

AIC: Akaike information criteria
BIC: Bayesian information criterion
DIC: deviance information criterion
FDR: false discovery rate
Fst: fixation index as a measure of genetic variance
GLM: generalized linear model
BMI: body mass index
INR: international normalized ratio based on prothrombin time
LD: linkage disequilibrium
MAF: minor allele frequency
NGS: next generation sequencing
PD: pharmacodynamics
PharmGKB: pharmacogenomics knowledge base
PK: pharmacokinetics
SNV: single nucleotide variant
SNP: single nucleotide polymorphism.

## References

[B9] Acland A. Agarwala R. Barrett T. Beck J. Benson D. A. Bollin C. (2013). Database resources of the national center for biotechnology information. Nucleic Acids Res. 41, D8–D20. 10.1093/nar/gks1189 23193264 PMC3531099

[B1] Allan P. Rettie E. Jones J. P. (2005). clinical and toxicological relevance of cyp2c9: drug-drug interactions and pharmacogenetics. Annu. Rev. Pharmacol. Toxicol. 45, 477–494. 10.1146/annurev.pharmtox.45.120403.095821 15822186

[B2] Baldi P. Long A. D. (2001). A Bayesian framework for the analysis of microarray expression data: regularized t-test and statistical inferences of gene changes. Bioinformatics 17, 509–519. 10.1093/bioinformatics/17.6.509 11395427

[B3] Bolger A. M. Lohse M. Usadel B. (2014). Trimmomatic: a flexible trimmer for Illumina sequence data. Bioinformatics 30, 2114–2120. 10.1093/bioinformatics/btu170 24695404 PMC4103590

[B4] Bonifaz-Peña V. Contreras A. V. Struchiner C. J. Roela R. A. Furuya-Mazzotti T. K. Chammas R.. (2014). Exploring the distribution of genetic markers of pharmacogenomics relevance in brazilian and mexican populations. PLoS ONE 9:e112640. 10.1371/journal.pone.0112640 25419701 PMC4242606

[B5] Carcao M. Re W. Ewenstein B. (2015). The role of previously untreated patient studies in understanding the development of FVIII inhibitors. Haemophilia 22, 22–31. 10.1111/hae.12790 26315604

[B6] Chen C. Mengersen K. L. Ickstadt K. Keith J. Clair L. Alston Anthony N. (2012). Case Studies in Bayesian Statistical Modelling and Analysis, 1st Edn. Brisbane, QLD: John Wiley.

[B7] Cheng R. Leung R. K. Chen Y. Pan Y. Tong Y. Li Z.. (2015). Virtual pharmacist: a platform for pharmacogenomics. PLoS ONE 10:e0141105. 10.1371/journal.pone.0141105 26496198 PMC4619711

[B8] Cingolani P. Platts A. Wang le L. Coon M. Nguyen T. Wang L.. (2012). A program for annotating and predicting the effects of single nucleotide polymorphisms, SnpEff: SNPs in the genome of *Drosophila melanogaster* strain w1118; iso-2; iso-3. Fly 6, 80–92. 10.4161/fly.19695 22728672 PMC3679285

[B10] Cruz-Correa O. F. León-Cachón R. B. R. Barrera-Saldaña H. A. Soberón X. (2017). Prediction of atorvastatin plasmatic concentrations in healthy volunteers using integrated pharmacogenetics sequencing. Pharmacogenomics 18, 121–131. 10.2217/pgs-2016-0072 27976987

[B11] Danese E. Montagnana M. Johnson J. A. Rettie A. E. Zambon C. F. Lubitz S. A.. (2012). Impact of the CYP4F2 p.V433M polymorphism on coumarin dose requirement: systematic review and meta-analysis. Clin. Pharmacol. Ther. 92, 746–756. 10.1038/clpt.2012.184 23132553 PMC3731755

[B12] Fricke-Galindo I. Jung-Cook H. LLerena A. López-López M. Scott S. Stein C. (2016). Interethnic variability of pharmacogenetic biomarkers in Mexican healthy volunteers: a report from the RIBEF (Ibero-American Network of Pharmacogenetics and Pharmacogenomics). Drug Metab. Pers. Ther. 31, 625–629. 10.1515/dmpt-2015-0030 26812836

[B13] Galanter J. M. Fernandez-Lopez J. C. Gignoux C. R. Barnholtz-Sloan J. Fernandez-Rozadilla C. Via M.. (2012). Development of a panel of genome-wide ancestry informative markers to study admixture throughout the Americas. PLoS Genet. 8:e1002554. 10.1371/journal.pgen.1002554 22412386 PMC3297575

[B14] Harrington D. J. Gorska R. Wheeler R. Davidson S. Murden S. Morse C.. (2008). Pharmacodynamic resistance to warfarin is associated with nucleotide substitutions in VKORC1. J. Thromb. Haemost. 6, 1663–1670. 10.1111/j.1538-7836.2008.03116.x 18680536

[B15] Heim M. H. Meyer U. A. (1992). Evolution of a highly polymorphic human cytochrome P450 gene cluster: CYP2D6. Genomics 14, 49–58. 10.1016/S0888-7543(05)80282-4 1358797

[B16] McKenna A. Hanna M. Banks E. Sivachenko A. Cibulskis K. Kernytsky A.. (2010). The genome analysis toolkit: a mapreduce framework for analyzing next-generation DNA sequencing data. Genome Res. 20, 1297–1303. 10.1101/gr.107524.110 20644199 PMC2928508

[B17] Oliveira-Paula G. H. Luizon M. R. Lacchini R. Fontana V. Silva P. S. Biagi C.. (2017). Gene-gene interactions among PRKCA, NOS3 and BDKRB2 polymorphisms affect the antihypertensive effects of enalapril. Basic Clin. Pharmacol. Toxicol. 120, 284–291. 10.1111/bcpt.12682 27696692

[B18] Pratt V. M. Zehnbauer B. Wilson J. A. Baak R. Babic N. Bettinotti M.. (2010). Characterization of 107 genomic DNA reference materials for CYP2D6, CYP2C19, CYP2C9, VKORC1, and UGT1A1: A GeT-RM and association for molecular pathology collaborative project. J. Mol. Diagnost. 12, 835–846. 10.2353/jmoldx.2010.100090 20889555 PMC2962854

[B19] Purcell S. Neale B. Todd-Brown K. Thomas L. Ferreira M. A. Bender D.. (2007). PLINK: a tool set for whole-genome association and population-based linkage analyses. Am. J. Hum. Genet. 81, 559–575. 10.1086/519795 17701901 PMC1950838

[B20] Relling M. V. Klein T. E. (2011). CPIC: Clinical Pharmacogenetics Implementation Consortium of the pharmacogenomics research network. Clin. Pharmacol. Ther. 89, 464–467. 10.1038/clpt.2010.279 21270786 PMC3098762

[B21] Rieder M. J. Reiner A. P. Gage B. F. Nickerson D. A. Eby C. S. McLeod H. L.. (2005). Effect of VKORC1 Haplotypes on transcriptional regulation and warfarin dose. N. Engl. J. Med. 352, 2285–2293. 10.1056/NEJMoa044503 15930419

[B22] Robinson J. T. Thorvaldsdóttir H. Winckler W. Guttman M. Lander E. S. Getz G.. (2011). Integrative genomics viewer. Nat. Biotech. 29, 24–26. 10.1038/nbt.1754 21221095 PMC3346182

[B23] Scott S. A. Khasawneh R. Peter I. Kornreich R. Desnick R. J. (2014). Combined CYP2C9, VKORC1 and CYP4F2 frequencies among racial and ethnic groups. Pharmacogenomics 11, 781–791. 10.2217/pgs.10.49 20504253 PMC2904527

[B24] Sebastiani P. Timofeev N. Dworkis D. A. Perls T. T. Steinberg M. H. (2009). Genome-wide association studies and the genetic dissection of complex traits. Am. J. Hematol. 84, 504–515. 10.1002/ajh.21440 19569043 PMC2895326

[B25] Sherry S. T. Ward M. H. Kholodov M. Baker J. Phan L. Smigielski E. M. (2001). dbSNP: the NCBI database of genetic variation. Nucleic Acids Res. 29, 308–311. 10.1093/nar/29.1.308 11125122 PMC29783

[B26] Soria J. M. Almasy L. Souto J. C. Sabaterlleal M. Fontcuberta J. Blangero J. (2009). The F7 gene and clotting factor VII levels: dissection of a human quantitative trait locus. 2005. Hum. Biol. 81, 853–867. 10.3378/027.081.0627 20504202

[B27] Tàssies D. Freire C. Pijoan J. Maragall S. Monteagudo J. Ordinas A.. (2002). Pharmacogenetics of acenocoumarol: cytochrome P450 CYP2C9 polymorphisms influence dose requirements and stability of anticoagulation. Haematologica 87, 1185–1191. 12414349

[B28] The 1000 Genomes Project Consortium Auton A. Brooks L. D. Durbin R. M. Garrison E. P. Kang H. M.. (2015). A global reference for human genetic variation. Nature 526, 68–74. 10.1038/nature15393 26432245 PMC4750478

[B29] Tong H. Y. Dávila-Fajardo C. L. Borobia A. M. Martínez-González L. J. Lubomirov R. Perea León L. M.. (2016). A new pharmacogenetic algorithm to predict the most appropriate dosage of acenocoumarol for stable anticoagulation in a mixed spanish population. PLoS ONE 11:e0150456. 10.1371/journal.pone.0150456 26977927 PMC4792430

[B30] Ufer M. (2005). Comparative pharmacokinetics of vitamin K antagonists. Clin. Pharmacokinet. 44, 1227–1246. 10.2165/00003088-200544120-00003 16372822

[B31] Van der Auwera G. A. Carneiro M. O. Hartl C. Poplin R. Del Angel G. Levy-Moonshine A.. (2013). From fastq data to high confidence variant calls: the genome analysis toolkit best practices pipeline. Curr. Protoc. Bioinformatics 43, 11.10.1–33. 10.1002/0471250953.bi1110s43 25431634 PMC4243306

[B32] van Leeuwen Y. Rosendaal F. R. van der Meer F. J. M. (2008). The relationship between maintenance dosages of three vitamin K antagonists: acenocoumarol, warfarin and phenprocoumon. Thromb. Res. 123, 225–230. 10.1016/j.thromres.2008.01.020 18407321

[B33] Villegas-Torres B. Sánchez-Girón F. Jaramillo-Villafuerte K. Soberón X. Gonzalez-Covarrubias V. (2015). Genotype frequencies of VKORC1 and CYP2C9 in Native and Mestizo populations from Mexico, potential impact for coumarin dosing. Gene 558, 235–240. 10.1016/j.gene.2014.12.068 25560189

[B34] Waldron D. (2016). Genetic variation: diving deep into the genome. Nat. Rev. Genet. 17, 716–717. 10.1038/nrg.2016.144 27773921

[B35] Wang D. Papp A. C. Sun X. (2015). Functional characterization of CYP2D6 enhancer polymorphisms. Hum. Mol. Genet. 24, 1556–1562. 10.1093/hmg/ddu566 25381333 PMC4381757

[B36] Whirl-Carrillo M. McDonagh E. M. Hebert J. M. Gong L. Sangkuhl K. Thorn C. F.. (2012). Pharmacogenomics knowledge for personalized medicine. Clin. Pharmacol. Ther. 92, 414–417. 10.1038/clpt.2012.96 22992668 PMC3660037

[B37] Zhang Y. de Boer A. Verhoef T. I. van der Meer F. J. Le Cessie S. Maitland-van der Zee A. H.. (2015). Comparison of dosing algorithms for acenocoumarol and phenprocoumon using clinical factors with the standard care in the Netherlands. Thromb. Res. 136, 94–100. 10.1016/j.thromres.2015.04.034 25971661

